# 4044 A Cardiovascular Health and Wellness Mobile Health Intervention Among Church-going African-Americans: Formative Evaluation of the FAITH! App

**DOI:** 10.1017/cts.2020.189

**Published:** 2020-07-29

**Authors:** LaPrincess Brewer, Ashok Kumbamu, Christina Smith, Sarah Jenkins, Clarence Jones, Sharonne Hayes, Christi A. Patten, Lisa A. Cooper, Lora Burke

**Affiliations:** 1 Mayo Clinic; 2 Mayo Clinic College of Medicine; 3 Hue-Man Partnership; 4 Johns Hopkins University School of Medicine; 5 University of Pittsburgh

## Abstract

OBJECTIVES/GOALS: To evaluate the FAITH! (Fostering African-American Improvement in Total Health) App mHealth lifestyle intervention by using post-intervention feedback obtained from participants in our intervention pilot study. METHODS/STUDY POPULATION: We used qualitative methods (focus groups) to elicit post-intervention feedback. Participants who completed the pilot study were recruited to one of two focus groups. Semi-structured focus groups were conducted to explore participants’ views on the app functionality, utility and satisfaction as well as its impact on healthy lifestyle change. Sessions were audio-recorded, transcribed verbatim and qualitative data were analyzed by systematic text condensation thematic analysis. RESULTS/ANTICIPATED RESULTS: Nine individuals participated (N = 4 and N = 5) in each of the two focus groups. Their mean age was 47.9 years (SD 12.1), 67% were women, and all had at least an education level of some college. Six overarching themes emerged from the data: (1) overall impression, (2) content usefulness (3) formatting, (4) implementation, (5) impact and (6) suggestions for improvement. Underpinning the themes was a high level of agreement that the intervention facilitated healthy behavioral change through cultural tailoring, multimedia education modules and social networking. Among the suggestions for improvement were streamlining of app self-monitoring features, personalization based on individual’s cardiovascular risk and attentiveness to nuanced cultural perspectives. DISCUSSION/SIGNIFICANCE OF IMPACT: This formative evaluation found the FAITH! App mHealth lifestyle intervention had high reported satisfaction and impact on the health-promoting behaviors of African-Americans, thereby improving their overall cardiovascular health. The findings provide further support for the acceptability of mHealth interventions among African-Americans. CONFLICT OF INTEREST DESCRIPTION: None.

